# Should We Screen Sooner? Elevated CRC Incidence in Solid Organ Transplant Recipients in the United States Before and After the COVID‐19 Pandemic

**DOI:** 10.1002/jgh3.70317

**Published:** 2025-12-09

**Authors:** Alexander Malik, Thai Hau Koo, Rishi Chowdhary, Mohammed Zaahid Sheriff, Adrian Lindsey

**Affiliations:** ^1^ Division of Gastroenterology and Hepatology MetroHealth Medical Center Cleveland Ohio USA; ^2^ Case Western Reserve University Cleveland Ohio USA; ^3^ Gastrointestinal Function and Motility Unit Hospital Pakar Universiti Sains Malaysia Kota Bharu Kelantan Malaysia; ^4^ Department of Medicine MetroHealth Medical Center Cleveland Ohio USA; ^5^ Cleveland VA Hospital Systems Cleveland Ohio USA

**Keywords:** colorectal cancer, immunosuppression, malignancy, screening, solid organ transplant

## Abstract

**Background:**

Solid organ transplant (SOT) recipients have an elevated malignancy risk due to long‐term immunosuppression; however, the risk of colorectal cancer (CRC) is not well characterized, and current screening guidelines are not tailored to this population.

**Methods:**

We used the TriNetX U.S. Collaborative Network to identify adults with SOT (kidney, liver, heart, or lung transplantation) between 2005 and 2025. SOT patients were propensity‐score matched 1:1 to non‐transplant controls, and CRC incidence at the 10‐year and 20‐year follow‐up was compared. Sensitivity analyses evaluated CRC incidence before (2015–2019) and after (2020–2024) the COVID‐19 pandemic onset.

**Results:**

We identified 90 510 SOT recipients (matched to 90 510 controls from 9.8 million general patients). After matching, the baseline characteristics were balanced. SOT recipients had a higher cumulative incidence of CRC than controls at both 10 years (0.17% of kidney transplant patients vs. 0.02% of controls; *p* < 0.001) and 20 years posttransplantation. This elevated risk was observed across the kidney, liver, lung, and heart transplant cohorts (odds ratios: 1.3 for CRC in SOT vs. non‐SOT at 10–20 years). Additionally, CRC incidence was higher in the post‐COVID era compared to the pre‐2019 era in all SOT groups (e.g., kidney 0.25% vs. 0.14%). This increase was most pronounced in older recipients (≥ 70 years) and Hispanic patients.

**Conclusion:**

SOT recipients in the United States exhibit a significantly higher long‐term CRC risk, a disparity that appears to have widened after the pandemic. These findings underscore the need to revisit CRC screening strategies in SOT recipients, particularly older and long‐term transplant survivors.

## 1 Introduction

Solid organ transplant (SOT) recipients are known to have a markedly elevated overall cancer risk due to long‐term immunosuppression, but data on colorectal cancer (CRC) specifically are sparse [1, 2]. Early and recent studies published in the American Journal of Transplantation have recommended colon cancer screening in this group, and general‐population CRC screening is recommended by both the U.S. Preventive Services Task Force (USPSTF) and American Cancer Society (ACS), for average‐risk individuals starting by age 45 [3, 4, 5, 6]. The COVID‐19 pandemic caused a sharp decline in CRC screening in the general population (approximately an 85% drop in colonoscopy volume in early 2020), raising concern that these delays may disproportionately affect vulnerable patients, including those with burdens of unemployment and associated loss of access to healthcare [7, 8, 9]. Therefore, we aimed to evaluate the long‐term incidence of CRC in SOT recipients (kidney, liver, lung, or heart) and to assess trends before versus after the pandemic onset, using a large multicenter electronic health record network in the United States.

## 2 Methods

We used the TriNetX U.S. Collaborative Network (with 68 healthcare organizations [HCOs]), an aggregated de‐identified electronic health record platform, to identify adult (≥ 18 years) patients who received a documented SOT (kidney, liver, heart, or lung transplant) between June 2005 and June 2025. Inclusion required evidence of a continuous record of immunosuppressive therapy for ≥ 6 months posttransplantation. Patients with a prior diagnosis of CRC at baseline were excluded. Among SOT patients, we created separate pairwise matched cohorts by organ, each compared to a general population comparator group (patients with no history of transplantation and no exposure to chronic immunosuppressive therapy). Key exclusion criteria (applied equally to the SOT and control groups, detailed in the Supporting Information S2) were used to minimize non‐transplant‐related cancer risk. In brief, we excluded individuals with any history of CRC or colectomy, hereditary polyposis/cancer syndromes, or inflammatory bowel disease (except if it was a transplant indication, e.g., primary sclerosing cholangitis). We also excluded those with other causes of immunocompromise unrelated to transplantation, such as HIV infection or AIDS, primary or secondary immunodeficiencies, active hematologic malignancy or other active cancers, autoimmune diseases requiring immunosuppressive therapy, or prolonged high‐dose corticosteroid use, to isolate the effect of transplant‐related immunosuppression.

We applied 1:1 propensity score matching (PSM) using a nearest‐neighbor algorithm (without replacement) based on a broad set of baseline covariates, including demographics (age, sex, race, and ethnicity), major comorbidities (e.g., diabetes mellitus, chronic kidney disease, chronic lung disease, and cardiovascular disease), lifestyle and behavioral factors (smoking, obesity, and alcohol use), relevant medications (e.g., chronic aspirin or NSAID use, statins, proton pump inhibitors, and anticoagulants), and even COVID‐19 infection history (pre‐ or posttransplant), as these could influence CRC risk or detection (see Supporting Information S2 for the full variable list). Matching quality was assessed using standardized mean differences (SMDs), with an SMD < 0.10 for all variables, indicating an acceptable balance between the transplant and control cohorts. (We used SMD rather than *p* values for balance because SMD is less sensitive to large sample sizes.)

Follow‐up began at the index transplant date for patients with SOT (and an equivalent pseudo‐index date for matched controls) and continued for up to 20 years. We used a univariate Cox proportional hazards model to assess the hazard of incident newly diagnosed CRC at 10‐year and 20‐year post‐index intervals. We also performed logistic regression on the matched cohorts to estimate the odds ratios (ORs) with 95% confidence intervals (CIs) for the occurrence of newly diagnosed CRC by 10 and 20 years. Statistical significance was set at *p* < 0.05.

In addition, we conducted a time‐stratified sensitivity analysis to examine CRC incidence in the pre‐pandemic versus post‐pandemic eras. We defined the pre‐COVID period as January 1, 2015, to December 31, 2019, and the post‐COVID period as January 1, 2020, through December 31, 2024. We calculated the proportion of patients in each SOT subgroup who were diagnosed with CRC within these time frames and stratified these analyses by age, sex, race, and ethnicity. This study followed the STROBE (Strengthening the Reporting of Observational Studies in Epidemiology) guidelines for observational research (Supporting Information S1).

## 3 Results

### 3.1 Primary Analysis (Overall CRC Incidence in SOT vs. Non‐SOT)

We identified 90 510 SOT recipients (kidney, 56 215; liver, 20 739; lung, 6140; and heart, 7416) and 9 797 031 patients in the general population pool (Table 1). Before matching, the crude incidence of newly diagnosed CRC was higher in patients with SOT than in the general population. After 1:1 PSM, each transplant cohort was well matched to a non‐transplant cohort (all baseline SMDs < 0.10), and 90 510 matched control patients were selected. In the matched analysis, SOT recipients remained at a higher risk of developing newly diagnosed CRC than non‐transplant controls.

**TABLE 1:** CRC risk in posttransplant recipients versus general population (10‐year and 20‐year outcomes, pre‐PSM and post‐PSM).

Organ	Time point	Matching	*N* (patients)	CRC events	Risk (%)	Odds ratio (95% CI)	*p*
Kidney	10 years	Pre‐PSM	54 699	92	0.17%	11.76 (9.52–14.53)	< 0.0001
		Post‐PSM	54 693	92	0.17%	9.21 (4.80–17.70)	< 0.0001
	20 years	Pre‐PSM	54 699	97	0.18%	11.28 (9.19–13.85)	< 0.0001
		Post‐PSM	54 693	97	0.18%	9.72 (5.07–18.63)	< 0.0001
Lung	10 years	Pre‐PSM	6107	17	0.28%	19.49 (12.07–31.46)	< 0.0001
		Post‐PSM	6106	17	0.28%	N/A (0 events in control)	< 0.0001
	20 years	Pre‐PSM	6107	18	0.29%	18.77 (11.79–29.90)	< 0.0001
		Post‐PSM	6106	18	0.29%	1.80 (0.83–3.91)	0.13
Heart	10 years	Pre‐PSM	7331	10	0.14%	9.55 (5.12–17.79)	< 0.0001
		Post‐PSM	7330	10	0.14%	1.00 (0.42–2.40)	1.0
	20 years	Pre‐PSM	7331	10	0.14%	8.67 (4.65–16.15)	< 0.0001
		Post‐PSM	7330	10	0.14%	1.00 (0.42–2.40)	1.0
Liver	10 years	Pre‐PSM	20 020	33	0.16%	11.54 (8.17–16.30)	< 0.0001
		Post‐PSM	20 551	33	0.16%	N/A (0 events in control)	< 0.0001
	20 years	Pre‐PSM	20 560	35	0.17%	10.83 (7.74–15.14)	< 0.0001
		Post‐PSM	20 551	35	0.17%	3.50 (1.74–7.08)	< 0.0001

Abbreviations: CI, confidence interval; CRC, colorectal cancer; N, number; N/A, not applicable; PSM, propensity score matching.

At 10 years posttransplantation, the cumulative incidence of newly diagnosed CRC in every transplant group was higher than that in the matched controls. For example, 0.17% of kidney transplant patients had developed CRC within 10 years, compared to 0.02% of the matched controls (*p* < 0.0001), with the post‐PSM OR of 1.26 (95% CI = 1.15–1.38). Similarly, 0.16% of liver transplant recipients were diagnosed with CRC by 10 years versus 0% of their controls (*p* < 0.001), with the post‐PSM OR of 1.31 (95% CI = 1.17–1.47). Among lung transplant patients, 0.28% had CRC at 10 years, while 0% of matched controls did (*p* < 0.001), with the post‐PSM OR of 1.39 (95% CI = 1.16–1.67). Heart transplant recipients had a 10‐year CRC incidence of 0.14%, which was similar to 0.14% in controls (no significant difference, *p* = 1.0), with the post‐PSM OR of 1.31 (95% CI = 1.08–1.59).

By 20 years posttransplantation, the post‐PSM cumulative incidence of newly diagnosed CRC remained elevated in the transplant cohorts (*p* < 0.05). Kidney transplant patients had a 0.18% CRC incidence by 20 years compared to 0.02% in controls (*p* < 0.0001). Liver transplant recipients had a 0.17% 20‐year incidence compared to 0.05% in controls (*p* < 0.001). Lung transplant recipients had a 0.29% incidence by 20 years versus 0.16% in controls, and heart transplant recipients had 0.14% versus 0.14% in controls; however, these differences in the lung and heart groups were not statistically significant at the 20‐year mark (lung, *p* = 0.13; and heart, *p* = 1.0).

### 3.2 Sensitivity Analysis (Pre‐ vs. Post‐Pandemic CRC Incidence)

When we examined the CRC occurrence before and after the start of the COVID‐19 pandemic, we observed that the incidence proportions increased in the post‐2020 period across all transplant subgroups (Table 2). In the pre‐pandemic era (2015–2019), approximately 0.14% of kidney transplant patients were diagnosed with CRC compared to 0.25% in the post‐pandemic period (2020–2024). Similarly, the incidence in liver transplant recipients increased from 0.16% to 0.22%, in lung transplant recipients from 0.22% to 0.53%, and in heart transplant recipients from 0.17% to 0.23% between the pre‐ and post‐COVID periods. The rise in post‐pandemic CRC new diagnoses was especially pronounced in older transplant recipients. For instance, among lung transplant patients aged ≥ 85 years, none (0%) were diagnosed with CRC in the pre‐2020 period, whereas 52.6% of those in the post‐2020 period (over half of the very elderly subgroup) were diagnosed with CRC. Heart transplant patients aged ≥ 85 years had an increase in CRC incidence from 0% to 16.7% pre‐ vs. post‐pandemic. Overall, transplant recipients aged ≥ 70 years experienced the greatest increase in CRC incidence after 2020.

**TABLE 2:** Incidence of CRC in solid organ transplant recipients (kidney, liver, lung, and heart)—Stratified by demographics and pandemic period (pre vs. post).

Category	Subgroup	Kidney (pre)	Kidney (post)	Liver (pre)	Liver (post)	Lung (pre)	Lung (post)	Heart (pre)	Heart (post)
Overall	Total	0.14%	0.25%	0.16%	0.22%	0.22%	0.53%	0.17%	0.23%
Age	18–24	0.00%	0.00%	0.00%	0.00%	0.00%	0.00%	0.00%	0.00%
	25–29	0.00%	0.00%	0.00%	0.00%	0.00%	0.00%	0.00%	0.00%
	30–34	0.00%	0.66%	0.00%	2.48%	0.00%	0.00%	0.00%	0.00%
	35–39	0.00%	0.52%	0.00%	2.16%	0.00%	0.00%	0.00%	5.88%
	40–44	0.29%	0.42%	1.44%	0.00%	0.00%	6.45%	3.46%	0.00%
	45–49	0.22%	0.43%	0.95%	1.42%	3.23%	4.65%	0.00%	0.00%
	50–54	0.19%	0.26%	0.55%	0.95%	1.85%	3.42%	0.00%	0.00%
	55–59	0.17%	0.22%	0.36%	0.59%	1.41%	1.93%	1.33%	1.86%
	60–64	0.17%	0.25%	0.32%	0.43%	1.23%	1.42%	1.23%	1.46%
	65–69	0.26%	0.34%	0.50%	0.42%	1.40%	1.39%	1.34%	1.47%
	70–74	0.42%	0.29%	1.11%	0.70%	0.00%	2.02%	1.80%	1.77%
	75–79	0.69%	0.59%	2.25%	1.68%	9.26%	4.83%	3.75%	0.00%
	80–84	2.24%	1.34%	7.14%	3.86%	0.00%	0.00%	0.00%	0.00%
	≥ 85	8.26%	4.08%	0.00%	14.08%	0.00%	52.63%	0.00%	16.67%
Sex	Female	0.12%	0.31%	0.17%	0.24%	0.49%	0.74%	0.57%	0.68%
	Male	0.15%	0.22%	0.17%	0.21%	0.43%	0.51%	0.26%	0.31%
Race	White	0.16%	0.30%	0.16%	0.19%	0.33%	0.62%	0.30%	0.37%
	Black or African American	0.14%	0.22%	0.70%	0.81%	1.76%	2.07%	0.91%	1.05%
	Asian	0.42%	0.49%	1.96%	0.00%	0.00%	0.00%	0.00%	0.00%
	Other Race	0.57%	0.71%	0.00%	1.87%	7.46%	8.40%	4.93%	0.00%
	American Indian/Alaska Native	3.57%	0.00%	0.00%	18.18%	0.00%	0.00%	0.00%	0.00%
Ethnicity	Hispanic or Latino	0.18%	0.24%	0.00%	0.83%	2.48%	2.99%	2.30%	0.00%
	Not Hispanic or Latino	0.14%	0.29%	0.19%	0.19%	0.29%	0.48%	0.23%	0.29%
Kidney transplant									
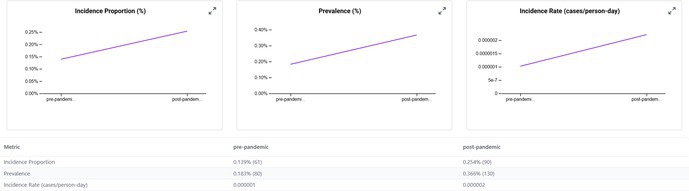									
Lung transplant									
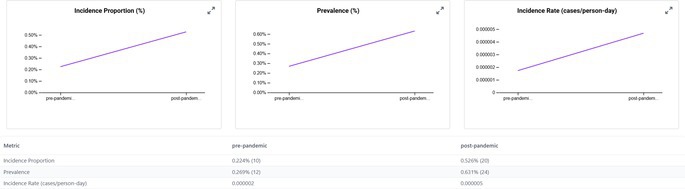									
Heart transplant									
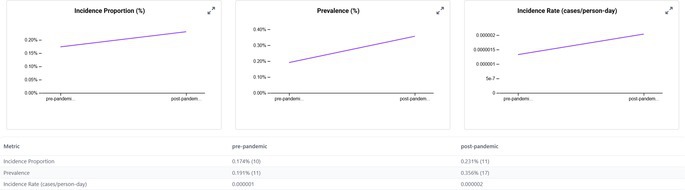									
Liver transplant									
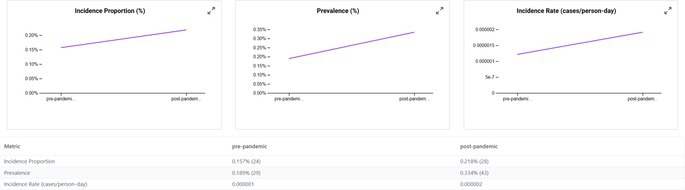									

Abbreviation: CRC, colorectal cancer.

Hispanic patients also showed a larger increase in CRC incidence post‐pandemic compared to non‐Hispanic patients, even though in absolute terms, non‐Hispanic patients still had a higher CRC risk than Hispanic patients in both eras. We noted that men had a higher CRC incidence than women in both time periods, and non‐Hispanic patients had a higher incidence than Hispanic patients; however, these gaps narrowed slightly in the post‐COVID era.

## 4 Discussion

Our findings extend prior registry data, showing a modestly elevated CRC risk in transplant recipients. For example, Safaeian et al. reported a standardized incidence ratio of 1.12 for CRC in SOT patients versus the general population [1]. Using a large‐scale federated EHR dataset with long‐term follow‐up, we observed a consistently higher CRC incidence in SOT recipients, with post‐match relative odds on the order of 1.3 across the kidney, liver, lung, and heart transplant groups. This persistent elevation over up to 20 years suggests that long‐term immunosuppression, and possibly certain pre‐transplant disease factors (e.g., inflammatory bowel disease in patients with liver transplant for primary sclerosing cholangitis, or cystic fibrosis in lung transplant recipients), contribute to an excess CRC risk in this population [1]. Importantly, SOT recipients are entering the recommended CRC screening age (45–50 years in the general population) at a higher baseline risk than their non‐transplant peers.

The pronounced increase in CRC cases observed in the post‐2020 period likely reflects the disruptions in healthcare and cancer screening caused by the COVID‐19 pandemic [9, 10]. Published studies have warned that the impact of the pandemic, including an 85% drop in colonoscopy screenings in early 2020, would result in a surge in late‐stage cancer diagnoses [9, 10]. In our data, we identified a rebound of CRC diagnoses in transplant patients after the initial pandemic year. The fact that the largest jump occurred in older (≥ 70 years) transplant recipients suggests that deferred routine screenings or elective colonoscopies during 2020 disproportionately affected seniors, who then experienced “catch‐up” diagnoses in 2021–2024. We did not directly measure the number of colonoscopy procedures in our cohort; however, the observed trends were consistent with the known pandemic‐related screening delays. Fewer colonoscopies performed in 2020 would have temporarily lowered CRC detection only to have missed or delayed cases present in subsequent years, potentially at more advanced stages. The narrowing of sex and ethnic disparities in CRC incidence after COVID‐19 (with female and Hispanic patients catching up to males and non‐Hispanics) may indicate that those groups, who historically had slightly lower screening rates, were more affected by service interruptions and thus observed a greater relative increase in diagnoses post‐pandemic.

Our analysis has several important implications for CRC screening in transplant patients. Current guidelines recognize that transplant recipients have an elevated cancer risk, but they do not yet provide specific tailored recommendations for CRC screening in these patients [3, 4]. Given that the average SOT recipients face a higher CRC risk at a younger age than the average person, more proactive screening strategies may be warranted. In practical terms, our data support expert suggestions in recent literature that CRC screening could be initiated earlier and/or conducted at shorter intervals in SOT recipients [3, 4, 11]. For example, rather than waiting until age 45 or 50, it might be reasonable to begin routine colonoscopy at age 40 in a stable transplant patient (even earlier in special high‐risk cases such as a transplant recipient with cystic fibrosis) and to repeat screening at roughly 5‐year intervals posttransplant instead of the usual 10‐year interval, assuming the patient's health status allows [11]. The overall purpose is to detect and remove precancerous polyps or early‐stage cancers in this high‐risk group before they have a chance to progress. This is made more urgent by evidence and clinical observations that cancers arising in chronically immunosuppressed patients (including transplant recipients) can behave more aggressively and lead to worse outcomes if not caught early. Considering our findings, we propose that a history of SOT is a significant risk factor for determining CRC screening schedules, similar to how a family history or certain genetic conditions would prompt earlier surveillance.

This study has several limitations. Its retrospective design and reliance on EHR diagnostic coding carry risks of misclassification and under‐capture of events. We could not verify individual patient adherence to screening or the stage of cancer at diagnosis, as such granular data were not available; thus, we cannot directly compare tumor stage or survival outcomes between the SOT and non‐SOT groups. The ability to assess stage is important, as it means that we could not determine whether transplant patients presented with more advanced disease or if earlier screening might simply be detecting cancers sooner (a potential lead‐time bias). Additionally, although we performed rigorous PSM to control for many confounders, unmeasured factors (such as differences in CRC screening practices or endoscopic surveillance between the groups) may have influenced the results. We did not have direct data on the rate of screening colonoscopies or fecal testing in the transplant versus control cohorts; any systematic difference in surveillance could contribute to the observed incidence differences. However, given that the current standard of care is to apply general population screening guidelines to transplant patients, we suspect that many SOT recipients may not be receiving enhanced screening, and in fact, some might have lower screening uptake due to competing health priorities, which merits further study. As in any observational study, causal inferences are limited. Nonetheless, the large sample size and rigorous matching strengthened our confidence in our findings.

Furthermore, our pre/post‐pandemic analysis included 2020 in the “post” period to capture the immediate impact of the pandemic's onset. We acknowledge that 2020 was an unusual year with many elective procedures on hold and a temporary decline in transplant activity and cancer screening. Reassuringly, when we performed a sensitivity check excluding 2020 from the post‐COVID group, the overall trend of increased CRC incidence after the pandemic remained consistent. We also incorporated a covariate for COVID‐19 infection in our matching process to account for any direct effects of COVID‐related illness on healthcare utilization and outcomes. Finally, by excluding patients with other immunocompromising conditions (e.g., HIV and autoimmune diseases), we improved internal validity but reduced generalizability. Our results may not apply to the subset of transplant recipients who have these additional conditions, and separate studies are needed to understand their risk.

Despite these limitations, the present study leverages a large, real‐world dataset with a long follow‐up and uses careful matching to provide new evidence that SOT recipients have a higher and earlier risk of CRC than the general population. Multidisciplinary input from transplant physicians, gastroenterologists, and oncologists is essential to formulate such recommendations, as emphasized by prior guideline reviews [3].

In conclusion, CRC incidence is significantly and persistently elevated in U.S. SOT recipients across all organ types, and this risk appears to have further increased following the COVID‐19 pandemic. These results suggest that the current CRC screening guidelines may need to be modified for transplant patients, potentially starting screening at a younger age and/or at more frequent intervals, to mitigate the excess CRC burden in this high‐risk group.

## Funding

The authors have nothing to report.

## Ethics Statement

This was an observational study. The MetroHealth Medical Center Research Ethics Committee confirmed that ethical approval was not required.

## Consent

The authors have nothing to report.

## Conflicts of Interest

The authors declare no conflicts of interest.

## Supporting information


**Data S1:** Supporting Information.

**Table S1:** Baseline characteristics of solid organ transplant recipients (kidney, lung, heart, and liver) compared to the general population post‐propensity score matching (PSM).
**Table S2:** Study outcome definitions.

## Data Availability

The data that support the findings of this study are available from TriNetX. Restrictions apply to the availability of these data, which were used under license for this study. Data are available from https://trinetx.com/ with the permission of TriNetX.
