# Supplementary material for: Should We Screen Sooner? Elevated CRC Incidence in Solid Organ Transplant Recipients in the United States Before and After the COVID‐19 Pandemic
Source: JGH Open. 2025 Dec 9;9(12):e70317. doi: 10.1002/jgh3.70317 (PMC12689456; doi:10.1002/jgh3.70317)
Supplement: Supplementary file 2 — Table S1: Baseline characteristics of solid organ transplant recipients (kidney, lung, heart, and liver) compared to the general population post‐propensity score matching (PSM). Table S2: Study outcome definitions. [file JGH3-9-e70317-s001.docx]

**Supplemental methods:**

***Inclusion Criteria***

The list of long-term immunosuppressive therapies/medications for ≥6 months post-index transplantation is as below:

azathioprine

Azathioprine, oral, 50 mg

Azathioprine, parenteral, 100 mg

basiliximab

belatacept

Injection, belatacept, 1 mg

cyclosporine

Cyclosporine

everolimus

Everolimus

Everolimus, oral, 0.25 mg

mycophenolate mofetil

Mycophenolate mofetil, oral, 250 mg

sirolimus

Sirolimus

Sirolimus, oral, 1 mg

Injection, sirolimus protein-bound particles, 1 mg

tacrolimus

Tacrolimus

Tacrolimus, immediate release, oral, 1 mg

Tacrolimus, parenteral, 5 mg

Tacrolimus, extended release, (envarsus xr), oral, 0.25 mg

Tacrolimus, extended release, (astagraf xl), oral, 0.1 mg

prednisone

prednisolone

Prednisone, immediate release or delayed release, oral, 1 mg

Prednisolone oral, per 5 mg

***Exclusion Criteria***

We excluded patients with non-transplant-related colorectal cancer (CRC) risk conditions to ensure etiologic specificity and isolate the effect of solid organ transplantation and chronic immunosuppression. Individuals with any prior diagnosis of CRC (including adenocarcinoma, carcinoma in situ, or advanced adenomas with high-grade dysplasia), prior colectomy (total or segmental), or any history of hereditary cancer syndromes (e.g., Lynch syndrome, familial adenomatous polyposis, MUTYH-associated polyposis, Peutz-Jeghers syndrome) were excluded at baseline to ensure incident CRC measurement. Similarly, patients with inflammatory bowel disease (IBD)—including Crohn’s disease and ulcerative colitis—were excluded unless IBD was the underlying indication for transplant (e.g., IBD-associated PSC in liver transplant recipients), to reduce residual confounding related to chronic inflammation-associated carcinogenesis.

Patients with known or suspected immunocompromised states unrelated to transplantation were excluded, including those with human immunodeficiency virus (HIV) infection or acquired immune deficiency syndrome (AIDS), primary or secondary immunodeficiencies (including common variable immunodeficiency, etc.), chronic corticosteroid use (>20 mg prednisone-equivalent for ≥30 days), chronic inflammation including autoimmune diseases requiring immunosuppressive therapy (e.g., systemic lupus erythematosus, rheumatoid arthritis, or inflammatory myopathies), hematologic malignancies (e.g., lymphoma, leukemia), or solid tumors (e.g., pancreatic, gastric, lung, breast, or gynecologic cancers) diagnosed prior to the index date. These exclusions were applied to both transplant and control cohorts to minimize non-transplant-related cancer risk.

Patients with prior or concurrent multiorgan transplantation (e.g., simultaneous liver-kidney, heart-lung, or pancreas-kidney) or re-transplantation events were excluded to ensure cohort homogeneity. Patients lacking evidence of continuous immunosuppressive therapy for ≥6 months post-index transplantation were excluded to account for incomplete immunologic exposure or early graft failure.

To minimize outcome confounding related to differential cancer surveillance or shortened life expectancy, we excluded individuals with advanced comorbid conditions likely to influence CRC detection, such as end-stage heart failure (ejection fraction <20%), metastatic non-colorectal cancer, or chronic mechanical ventilation dependence. Pregnant or postpartum individuals (defined as pregnancy or delivery within 12 months before or after index) were excluded due to differences in metabolic, hormonal, and surveillance pathways that affect gastrointestinal function, medication exposure, and neoplastic progression. Additionally, patients under 18 years of age were excluded to standardize adult transplant-related risk profiles and avoid inclusion of pediatric-onset conditions with distinct CRC risk mechanisms.

***Propensity Score Matching (PSM)***

We applied a 1 to 1 propensity score matching using nearest-neighbor greedy algorithms. Each component of the risk factors / confounders of CRC incidence, was individually matched for (i.e. Age at Index Event, demographics [age, sex, race, and ethnicity], comorbid conditions based on Elixhauser/Charlson variables (to control overall disease burden), such as diabetes mellitus type 2, chronic kidney disease (CKD), chronic obstructive pulmonary disease (COPD), congestive heart failure (CHF), hypertension, obesity (BMI ≥30), or alcohol/tobacco use (current or former); medication exposures including chronic aspirin or NSAID use, proton pump inhibitors (PPIs), statins, and anticoagulants, which may modulate gastrointestinal tumorigenesis risk; psychiatric or behavioral comorbidities including major depressive disorder, generalized anxiety disorder, schizophrenia, bipolar disorder, personality disorders, post-traumatic stress disorder (PTSD), attention-deficit/hyperactivity disorder (ADHD), and chronic pain syndromes, as these have been associated with lower screening adherence, and substance use behaviors; COVID-19-specific variables including SARS-CoV-2 infection status (pre- or post-index), and documentation of long COVID diagnosis, which may confound healthcare-seeking behavior, medication exposure, or cancer detection timing.

Covariate balance between matched cohorts was assessed using standardized mean differences (SMD), with an SMD <0.1 interpreted as an acceptable level of covariate balance. All matching and balance diagnostics were conducted using the TriNetX Analytics platform. Only patients with complete follow-up data for at least 12 months after index were retained for primary outcome assessment to ensure adequate observation time for CRC incidence analysis.

***Data Providers***
The TriNetX USA network is a de-identified, longitudinal data source that includes both inpatient and outpatient electronic health records (EHR) from participating healthcare organizations (HCO) across the United States. These patient-level data were sourced from a global federated health research network with almost real-time updates (typically updated every 2-4 weeks). Network members included academic medical centers, integrated delivery networks, specialty hospitals, and large specialty physician practices. The dataset contained detailed clinical information, including demographics, diagnoses, prescribed medications, laboratory test results, vital signs, and procedures performed for each medical encounter and day of hospital stay within the network.

***Data Privacy and Internal Review Board***

TriNetX is structured to allow patient-level analyses while reporting only population-level data to maintain subject anonymity. TriNetX treats all counts between one to ten as equivalent to further maintain anonymity. Because of the de-identified and aggregated nature of the data in the database at the standard defined in Section §164.514(a) of the HIPAA Privacy Rule, the Metrohealth Medical Center Institutional Review Board has deemed studies using the TriNetX database exempt from requiring IRB approval.
 
***Data Elements***
Data elements are those in the fixed fields of the EHR (i.e., demographics, laboratory results, vitals, diagnoses, procedures, and prescribed medications), data captured via text mining of progress notes and other documents within the patient’s record, and additional data linked at the patient level (e.g., mortality).
 
TriNetX typically receives data from HCOs and other data providers in one of the two ways.

1. TriNetX ingests data directly from an HCO research repository (e.g., i2b2) into the TriNetX environment.
2. An HCO or data provider sends TriNetX data extracts in the form of CSV files

All patient data in the TriNetX network were harmonized with standard terminologies. Diagnoses in the patients’ medical records were coded according to the International Classification of Diseases, Tenth Revision, Clinical Modification (ICD-10-CM) diagnosis codes. Procedures were defined by the Current Procedural Terminology (CPT), Healthcare Common Procedure Coding System (HCPCS), and International Classification of Diseases, Tenth Revision, Procedure Coding System (ICD-10-PCS) procedure codes. Medication orders were defined by or mapped to RxNorm Ingredient, CPT, HCPCS, and ICD-10-PCS medication codes. Laboratory test results were defined using Logical Observation Identifiers Names and Codes (LOINC).
 
The data are then transformed into a proprietary data schema. This transformation process includes an extensive data quality assessment that includes ‘data cleaning,’ which rejects records that do not meet TriNetX quality standards. The data elements in TriNetX include but are not limited to

- **Demographic data** included elements such as age, gender, race, ethnicity, and US Census region. The race data within the TriNetX data model are consistent with the HL7 CDC Version 1 standards, which include **American Indian or Alaska Native, Black or African American, Native Hawaiian or Other Pacific Islander, and White. Race and ethnicity data are sourced directly from the provider HCOs and may be either self-reported or**observed by providers in patients’ EHRs.
- **Encounter data** included elements such as start date, end date, and encounter type (ambulatory, emergency room, inpatient, home health, inpatient non-acute, observation, pre-admission, short stay, and virtual).
- **Diagnosis data** were mapped to the International Classification of Diseases, Tenth Revision, Clinical Modification (ICD-10-CM) diagnosis codes, including diagnosis codes, dates, and whether the diagnosis was indicated as primary, secondary, or unknown. Diagnoses may also be indicated as admitting diagnoses or reasons for visit.
- **Procedure data** were defined by the Current Procedural Terminology (CPT), Healthcare Common Procedure Coding System (HCPCS), and International Classification of Diseases, Tenth Revision, Procedure Coding System (ICD-10-PCS) procedure codes, including code, date, and whether the procedure was indicated as primary, secondary, or unknown.
- **Medication data** were defined by or mapped to RxNorm Ingredient codes, CPT, HCPCS, and ICD-10-PCS medication codes, including code, start date, route of administration, brand, strength, quantity dispensed, and supply days. TriNetX identifies active prescriptions administered by a provider, reported by the pharmacy, or reported by the patient during a medical encounter.
- **Laboratory data** were mapped to Logical Observation Identifiers Names and Codes (LOINC) or TriNetX custom codes, and included code, date recorded, lab results (numeric or positive/negative/unknown), and units of measure.
- **Vital signs** are mapped to LOINC or TriNetX custom codes and include the code, date, value, and units of measure.
- There are also oncology- and genomic-specific data types that cover tumor morphology, cancer staging, oncology treatments, and genetic variants.

Only the data elements listed above are reported as available and appropriate. Any data elements derived by TriNetX or natural language processing are indicated in the data tables.

**Supplemental Tables:**

**Table S1.** Baseline characteristics of solid organ transplant recipients (kidney, lung, heart, and liver) compared to the general population post–propensity score matching (PSM).

| **Kidney Transplant Cohort**   \| **Cohort 1 and cohort 2 patient count before and after propensity score matching** \| \| \| \| \| --- \| --- \| --- \| --- \| \|  \| Cohort \| Patient count before matching \| Patient count after matching \| \|  \| 1 - Cohort 1: kidney transplant \| 56,215 \| 54,693 \| \|  \| 2 - Cohort 5: General Non-Transplant Population \| 9,797,031 \| 54,693 \| | |
| --- | --- | --- | --- | --- | --- | --- | --- | --- | --- | --- | --- | --- | --- | --- | --- | --- | --- |
| \| **Cohort 1 (N = 56,215) and cohort 2 (N = 9,797,031) characteristics before propensity score matching** \| \| \| \| \| \| \| \| \| \| \| --- \| --- \| --- \| --- \| --- \| --- \| --- \| --- \| --- \| --- \| \|  \| **Demographics** \| \| \| \| \| \| \| \| \| \|  \|  \| Cohort \| \|  \| Mean ± SD \| Patients \| % of Cohort \| P-Value \| Std diff. \| \|  \|  \| 1 2 \|  \| Age at Index \| 54.3 +/- 15.6 50.2 +/- 20.5 \| 54,699 9,627,662 \| 100% 100% \| <0.001 \| 0.224 \| \|  \|  \| 1 2 \|  \| White \|  \| 28,101 6,461,979 \| 51.4% 67.1% \| <0.001 \| 0.325 \| \|  \|  \| 1 2 \|  \| Female \|  \| 22,412 5,493,065 \| 41.0% 57.1% \| <0.001 \| 0.326 \| \|  \|  \| 1 2 \|  \| Black or African American \|  \| 13,404 1,386,513 \| 24.5% 14.4% \| <0.001 \| 0.257 \| \|  \| **Diagnosis** \| \| \| \| \| \| \| \| \| \|  \|  \| Cohort \| \|  \| Mean ± SD \| Patients \| % of Cohort \| P-Value \| Std diff. \| \|  \|  \| 1 2 \|  \| Tobacco use \|  \| 3,297 1,058,057 \| 6.0% 11.0% \| <0.001 \| 0.179 \| \|  \|  \| 1 2 \|  \| Alcohol use \|  \| 631 189,956 \| 1.2% 2.0% \| <0.001 \| 0.066 \| \|  \|  \| 1 2 \|  \| Crohn's disease \|  \| 40 1,941 \| 0.1% 0.0% \| <0.001 \| 0.025 \| \|  \|  \| 1 2 \|  \| Ulcerative colitis \|  \| 53 1,490 \| 0.1% 0.0% \| <0.001 \| 0.034 \| \|  \|  \| 1 2 \|  \| Type 2 diabetes mellitus \|  \| 19,324 1,114,391 \| 35.3% 11.6% \| <0.001 \| 0.584 \| \|  \|  \| 1 2 \|  \| Obesity \|  \| 8,432 1,311,214 \| 15.4% 13.6% \| <0.001 \| 0.051 \| | \| **Cohort 1 (N = 54,693) and cohort 2 (N = 54,693) characteristics after propensity score matching** \| \| \| \| \| \| \| \| \| \| \| --- \| --- \| --- \| --- \| --- \| --- \| --- \| --- \| --- \| --- \| \|  \| **Demographics** \| \| \| \| \| \| \| \| \| \|  \|  \| Cohort \| \|  \| Mean ± SD \| Patients \| % of Cohort \| P-Value \| Std diff. \| \|  \|  \| 1 2 \|  \| Age at Index \| 54.3 +/- 15.6 54.3 +/- 15.6 \| 54,693 54,693 \| 100% 100% \| 0.963 \| <0.001 \| \|  \|  \| 1 2 \|  \| White \|  \| 28,098 28,095 \| 51.4% 51.4% \| 0.986 \| <0.001 \| \|  \|  \| 1 2 \|  \| Female \|  \| 22,410 22,400 \| 41.0% 41.0% \| 0.951 \| <0.001 \| \|  \|  \| 1 2 \|  \| Black or African American \|  \| 13,403 13,407 \| 24.5% 24.5% \| 0.978 \| <0.001 \| \|  \| **Diagnosis** \| \| \| \| \| \| \| \| \| \|  \|  \| Cohort \| \|  \| Mean ± SD \| Patients \| % of Cohort \| P-Value \| Std diff. \| \|  \|  \| 1 2 \|  \| Tobacco use \|  \| 3,297 3,298 \| 6.0% 6.0% \| 0.990 \| <0.001 \| \|  \|  \| 1 2 \|  \| Alcohol use \|  \| 631 635 \| 1.2% 1.2% \| 0.910 \| 0.001 \| \|  \|  \| 1 2 \|  \| Crohn's disease \|  \| 38 33 \| 0.1% 0.1% \| 0.553 \| 0.004 \| \|  \|  \| 1 2 \|  \| Ulcerative colitis \|  \| 49 46 \| 0.1% 0.1% \| 0.758 \| 0.002 \| \|  \|  \| 1 2 \|  \| Type 2 diabetes mellitus \|  \| 19,318 19,322 \| 35.3% 35.3% \| 0.980 \| <0.001 \| \|  \|  \| 1 2 \|  \| Obesity \|  \| 8,428 8,426 \| 15.4% 15.4% \| 0.987 \| <0.001 \| |
| **Lung Transplant Cohort**   \| **Cohort 1 and cohort 2 patient count before and after propensity score matching** \| \| \| \| \| --- \| --- \| --- \| --- \| \|  \| Cohort \| Patient count before matching \| Patient count after matching \| \|  \| 1 - Cohort 2: lung transplant \| 6,140 \| 6,106 \| \|  \| 2 - Cohort 5: General Non-Transplant Population \| 9,797,031 \| 6,106 \| | |
| \| **Cohort 1 (N = 6,140) and cohort 2 (N = 9,797,031) characteristics before propensity score matching** \| \| \| \| \| \| \| \| \| \| \| --- \| --- \| --- \| --- \| --- \| --- \| --- \| --- \| --- \| --- \| \|  \| **Demographics** \| \| \| \| \| \| \| \| \| \|  \|  \| Cohort \| \|  \| Mean ± SD \| Patients \| % of Cohort \| P-Value \| Std diff. \| \|  \|  \| 1 2 \|  \| Age at Index \| 58.1 +/- 14.4 50.2 +/- 20.5 \| 6,107 9,627,662 \| 100% 100% \| <0.001 \| 0.447 \| \|  \|  \| 1 2 \|  \| White \|  \| 4,191 6,461,979 \| 68.6% 67.1% \| 0.012 \| 0.032 \| \|  \|  \| 1 2 \|  \| Female \|  \| 2,760 5,493,065 \| 45.2% 57.1% \| <0.001 \| 0.239 \| \|  \|  \| 1 2 \|  \| Black or African American \|  \| 711 1,386,513 \| 11.6% 14.4% \| <0.001 \| 0.082 \| \|  \| **Diagnosis** \| \| \| \| \| \| \| \| \| \|  \|  \| Cohort \| \|  \| Mean ± SD \| Patients \| % of Cohort \| P-Value \| Std diff. \| \|  \|  \| 1 2 \|  \| Tobacco use \|  \| 537 1,058,057 \| 8.8% 11.0% \| <0.001 \| 0.074 \| \|  \|  \| 1 2 \|  \| Alcohol use \|  \| 140 189,956 \| 2.3% 2.0% \| 0.073 \| 0.022 \| \|  \|  \| 1 2 \|  \| Crohn's disease \|  \| 10 1,941 \| 0.2% 0.0% \| <0.001 \| 0.047 \| \|  \|  \| 1 2 \|  \| Ulcerative colitis \|  \| 10 1,490 \| 0.2% 0.0% \| <0.001 \| 0.050 \| \|  \|  \| 1 2 \|  \| Type 2 diabetes mellitus \|  \| 1,568 1,114,391 \| 25.7% 11.6% \| <0.001 \| 0.368 \| \|  \|  \| 1 2 \|  \| Obesity \|  \| 1,013 1,311,214 \| 16.6% 13.6% \| <0.001 \| 0.083 \| | \| **Cohort 1 (N = 6,106) and cohort 2 (N = 6,106) characteristics after propensity score matching** \| \| \| \| \| \| \| \| \| \| \| --- \| --- \| --- \| --- \| --- \| --- \| --- \| --- \| --- \| --- \| \|  \| **Demographics** \| \| \| \| \| \| \| \| \| \|  \|  \| Cohort \| \|  \| Mean ± SD \| Patients \| % of Cohort \| P-Value \| Std diff. \| \|  \|  \| 1 2 \| AI \| Age at Index \| 58.1 +/- 14.4 58.0 +/- 14.8 \| 6,106 6,106 \| 100% 100% \| 0.843 \| 0.004 \| \|  \|  \| 1 2 \| 2106-3 \| White \|  \| 4,190 4,239 \| 68.6% 69.4% \| 0.338 \| 0.017 \| \|  \|  \| 1 2 \| F \| Female \|  \| 2,760 2,705 \| 45.2% 44.3% \| 0.317 \| 0.018 \| \|  \|  \| 1 2 \| 2054-5 \| Black or African American \|  \| 711 676 \| 11.6% 11.1% \| 0.318 \| 0.018 \| \|  \| **Diagnosis** \| \| \| \| \| \| \| \| \| \|  \|  \| Cohort \| \|  \| Mean ± SD \| Patients \| % of Cohort \| P-Value \| Std diff. \| \|  \|  \| 1 2 \| F17 \| Tobacco use \|  \| 537 557 \| 8.8% 9.1% \| 0.526 \| 0.011 \| \|  \|  \| 1 2 \| F10.1 \| Alcohol use \|  \| 140 147 \| 2.3% 2.4% \| 0.676 \| 0.008 \| \|  \|  \| 1 2 \| K50 \| Crohn's disease \|  \| 10 10 \| 0.2% 0.2% \| 1 \| <0.001 \| \|  \|  \| 1 2 \| K51 \| Ulcerative colitis \|  \| 10 10 \| 0.2% 0.2% \| 1 \| <0.001 \| \|  \|  \| 1 2 \| E11 \| Type 2 diabetes mellitus \|  \| 1,567 1,549 \| 25.7% 25.4% \| 0.709 \| 0.007 \| \|  \|  \| 1 2 \| E66 \| Obesity \|  \| 1,012 1,011 \| 16.6% 16.6% \| 0.981 \| <0.001 \| |
| **Heart Transplant Cohort**   \| **Cohort 1 and cohort 2 patient count before and after propensity score matching** \| \| \| \| \| --- \| --- \| --- \| --- \| \|  \| Cohort \| Patient count before matching \| Patient count after matching \| \|  \| 1 - Cohort 3: heart transplant \| 7,416 \| 7,330 \| \|  \| 2 - Cohort 5: General Non-Transplant Population \| 9,597,767 \| 7,330 \| | |
| \| **Cohort 1 (N = 7,416) and cohort 2 (N = 9,597,767) characteristics before propensity score matching** \| \| \| \| \| \| \| \| \| \| \| --- \| --- \| --- \| --- \| --- \| --- \| --- \| --- \| --- \| --- \| \|  \| **Demographics** \| \| \| \| \| \| \| \| \| \|  \|  \| Cohort \| \|  \| Mean ± SD \| Patients \| % of Cohort \| P-Value \| Std diff. \| \|  \|  \| 1 2 \|  \| Age at Index \| 55.2 +/- 18.1 50.1 +/- 20.6 \| 7,331 9,428,398 \| 100% 100% \| <0.001 \| 0.267 \| \|  \|  \| 1 2 \|  \| White \|  \| 4,280 6,328,323 \| 58.4% 67.1% \| <0.001 \| 0.181 \| \|  \|  \| 1 2 \|  \| Female \|  \| 2,179 5,372,661 \| 29.7% 57.0% \| <0.001 \| 0.572 \| \|  \|  \| 1 2 \|  \| Black or African American \|  \| 1,303 1,343,753 \| 17.8% 14.3% \| <0.001 \| 0.096 \| \|  \| **Diagnosis** \| \| \| \| \| \| \| \| \| \|  \|  \| Cohort \| \|  \| Mean ± SD \| Patients \| % of Cohort \| P-Value \| Std diff. \| \|  \|  \| 1 2 \|  \| Tobacco use \|  \| 581 1,033,175 \| 7.9% 11.0% \| <0.001 \| 0.104 \| \|  \|  \| 1 2 \|  \| Alcohol use \|  \| 188 187,382 \| 2.6% 2.0% \| <0.001 \| 0.039 \| \|  \|  \| 1 2 \|  \| Crohn's disease \|  \| 10 1,885 \| 0.1% 0.0% \| <0.001 \| 0.042 \| \|  \|  \| 1 2 \|  \| Ulcerative colitis \|  \| 10 1,424 \| 0.1% 0.0% \| <0.001 \| 0.044 \| \|  \|  \| 1 2 \|  \| Type 2 diabetes mellitus \|  \| 2,414 1,076,665 \| 32.9% 11.4% \| <0.001 \| 0.536 \| \|  \|  \| 1 2 \|  \| Obesity \|  \| 1,266 1,262,790 \| 17.3% 13.4% \| <0.001 \| 0.108 \| | \| **Cohort 1 (N = 7,330) and cohort 2 (N = 7,330) characteristics after propensity score matching** \| \| \| \| \| \| \| \| \| \| \| --- \| --- \| --- \| --- \| --- \| --- \| --- \| --- \| --- \| --- \| \|  \| **Demographics** \| \| \| \| \| \| \| \| \| \|  \|  \| Cohort \| \|  \| Mean ± SD \| Patients \| % of Cohort \| P-Value \| Std diff. \| \|  \|  \| 1 2 \|  \| Age at Index \| 55.3 +/- 18.1 55.2 +/- 18.1 \| 7,330 7,330 \| 100% 100% \| 0.931 \| 0.001 \| \|  \|  \| 1 2 \|  \| White \|  \| 4,279 4,275 \| 58.4% 58.3% \| 0.947 \| 0.001 \| \|  \|  \| 1 2 \|  \| Female \|  \| 2,179 2,177 \| 29.7% 29.7% \| 0.971 \| 0.001 \| \|  \|  \| 1 2 \|  \| Black or African American \|  \| 1,303 1,303 \| 17.8% 17.8% \| 1 \| <0.001 \| \|  \| **Diagnosis** \| \| \| \| \| \| \| \| \| \|  \|  \| Cohort \| \|  \| Mean ± SD \| Patients \| % of Cohort \| P-Value \| Std diff. \| \|  \|  \| 1 2 \|  \| Tobacco use \|  \| 580 579 \| 7.9% 7.9% \| 0.976 \| 0.001 \| \|  \|  \| 1 2 \|  \| Alcohol use \|  \| 188 191 \| 2.6% 2.6% \| 0.876 \| 0.003 \| \|  \|  \| 1 2 \|  \| Crohn's disease \|  \| 10 10 \| 0.1% 0.1% \| 1 \| <0.001 \| \|  \|  \| 1 2 \|  \| Ulcerative colitis \|  \| 10 10 \| 0.1% 0.1% \| 1 \| <0.001 \| \|  \|  \| 1 2 \|  \| Type 2 diabetes mellitus \|  \| 2,413 2,411 \| 32.9% 32.9% \| 0.972 \| 0.001 \| \|  \|  \| 1 2 \|  \| Obesity \|  \| 1,266 1,267 \| 17.3% 17.3% \| 0.983 \| <0.001 \| |
| **Liver Transplant Cohort**   \| **Cohort 1 and cohort 2 patient count before and after propensity score matching** \| \| \| \| \| --- \| --- \| --- \| --- \| \|  \| Cohort \| Patient count before matching \| Patient count after matching \| \|  \| 1 - Cohort 4: liver transplant \| 20,739 \| 20,551 \| \|  \| 2 - Cohort 5: General Non-Transplant Population \| 9,797,031 \| 20,551 \| | |
| \| **Cohort 1 (N = 20,739) and cohort 2 (N = 9,797,031) characteristics before propensity score matching** \| \| \| \| \| \| \| \| \| \| \| --- \| --- \| --- \| --- \| --- \| --- \| --- \| --- \| --- \| --- \| \|  \| **Demographics** \| \| \| \| \| \| \| \| \| \|  \|  \| Cohort \| \|  \| Mean ± SD \| Patients \| % of Cohort \| P-Value \| Std diff. \| \|  \|  \| 1 2 \|  \| Age at Index \| 56.2 +/- 15.2 50.2 +/- 20.5 \| 20,560 9,627,662 \| 100% 100% \| <0.001 \| 0.334 \| \|  \|  \| 1 2 \|  \| White \|  \| 14,365 6,461,979 \| 69.9% 67.1% \| <0.001 \| 0.059 \| \|  \|  \| 1 2 \|  \| Female \|  \| 7,886 5,493,065 \| 38.4% 57.1% \| <0.001 \| 0.381 \| \|  \|  \| 1 2 \|  \| Black or African American \|  \| 1,817 1,386,513 \| 8.8% 14.4% \| <0.001 \| 0.174 \| \|  \| **Diagnosis** \| \| \| \| \| \| \| \| \| \|  \|  \| Cohort \| \|  \| Mean ± SD \| Patients \| % of Cohort \| P-Value \| Std diff. \| \|  \|  \| 1 2 \|  \| Tobacco use \|  \| 1,894 1,058,057 \| 9.2% 11.0% \| <0.001 \| 0.059 \| \|  \|  \| 1 2 \|  \| Alcohol abuse \|  \| 1,710 189,956 \| 8.3% 2.0% \| <0.001 \| 0.290 \| \|  \|  \| 1 2 \|  \| Crohn's disease \|  \| 30 1,941 \| 0.1% 0.0% \| <0.001 \| 0.044 \| \|  \|  \| 1 2 \|  \| Ulcerative colitis \|  \| 76 1,490 \| 0.4% 0.0% \| <0.001 \| 0.081 \| \|  \|  \| 1 2 \|  \| Type 2 diabetes mellitus \|  \| 5,728 1,114,391 \| 27.9% 11.6% \| <0.001 \| 0.418 \| \|  \|  \| 1 2 \|  \| Obesity \|  \| 2,831 1,311,214 \| 13.8% 13.6% \| 0.530 \| 0.004 \| | \| **Cohort 1 (N = 20,551) and cohort 2 (N = 20,551) characteristics after propensity score matching** \| \| \| \| \| \| \| \| \| \| \| --- \| --- \| --- \| --- \| --- \| --- \| --- \| --- \| --- \| --- \| \|  \| **Demographics** \| \| \| \| \| \| \| \| \| \|  \|  \| Cohort \| \|  \| Mean ± SD \| Patients \| % of Cohort \| P-Value \| Std diff. \| \|  \|  \| 1 2 \|  \| Age at Index \| 56.2 +/- 15.2 56.2 +/- 15.2 \| 20,551 20,551 \| 100% 100% \| 0.976 \| <0.001 \| \|  \|  \| 1 2 \|  \| White \|  \| 14,357 14,369 \| 69.9% 69.9% \| 0.897 \| 0.001 \| \|  \|  \| 1 2 \|  \| Female \|  \| 7,886 7,902 \| 38.4% 38.5% \| 0.871 \| 0.002 \| \|  \|  \| 1 2 \|  \| Black or African American \|  \| 1,817 1,798 \| 8.8% 8.7% \| 0.741 \| 0.003 \| \|  \| **Diagnosis** \| \| \| \| \| \| \| \| \| \|  \|  \| Cohort \| \|  \| Mean ± SD \| Patients \| % of Cohort \| P-Value \| Std diff. \| \|  \|  \| 1 2 \|  \| Tobacco use \|  \| 1,894 1,914 \| 9.2% 9.3% \| 0.734 \| 0.003 \| \|  \|  \| 1 2 \|  \| Alcohol use \|  \| 1,709 1,711 \| 8.3% 8.3% \| 0.972 \| <0.001 \| \|  \|  \| 1 2 \|  \| Crohn's disease \|  \| 29 27 \| 0.1% 0.1% \| 0.789 \| 0.003 \| \|  \|  \| 1 2 \|  \| Ulcerative colitis \|  \| 67 66 \| 0.3% 0.3% \| 0.931 \| 0.001 \| \|  \|  \| 1 2 \|  \| Type 2 diabetes mellitus \|  \| 5,721 5,741 \| 27.8% 27.9% \| 0.826 \| 0.002 \| \|  \|  \| 1 2 \|  \| Obesity \|  \| 2,829 2,846 \| 13.8% 13.8% \| 0.808 \| 0.002 \| |

**Table S2:** Study Outcome Definitions.

| Colorectal Cancer | | | | |
| --- | --- | --- | --- | --- |
|  | **Outcome definition** | | | |
|  | | Diagnosis | UMLS:ICD10CM:C18 | Malignant neoplasm of colon |
|  | | Diagnosis | UMLS:ICD10CM:C19 | Malignant neoplasm of rectosigmoid junction |
|  | | Diagnosis | UMLS:ICD10CM:C20 | Malignant neoplasm of rectum |
|  | | GlobalOncology | UMLS:ICDO3:C18 | Colon |
|  | | GlobalOncology | UMLS:ICDO3:C19 | Rectosigmoid junction |
|  | | GlobalOncology | UMLS:ICDO3:C20 | Rectum |
|  | | Diagnosis | UMLS:ICD10CM:Z85.03 | Personal history of malignant neoplasm of large intestine |
|  | | Diagnosis | UMLS:ICD10CM:Z85.04 | Personal history of malignant neoplasm of rectum, rectosigmoid junction, and anus |
|  | **Settings for the performed analyses** | | | |
|  | | Risk analysis | | including patients with outcome prior to the time window |
|  | | Kaplan - Meier survival analysis | | including patients with outcome prior to the time window |
